# Meetings of Societies

**Published:** 1939

**Authors:** 


### Meetings of Societies

### Bristol Medico-Chirurgical Society.

SIXTY-SIXTH SESSION, 1938–1939.

The last meeting of the session was held in the Physics Lecture Theatre, Royal Fort, on Wednesday, 10th May, 1939, at 8.30 p.m. The President, Dr. Leonard Moore, was in the chair and 45 members were present. Professor Hewer showed pathological specimens.

Mr. E. Watson-Williams opened a discussion on "**Spasmodic Rhinorrhœa,**" particularly the non-seasonal types: his paper appeared on page 109 of our last issue. It was followed by the best discussion of the session, to which no fewer than thirteen members contributed: The President, Mr. Wright, Dr. Foss, Professor Nixon, Mr. Angell James, Dr. Ling, Mr. Rayner, Mr. Palin, Dr. Orr-Ewing, Dr. Soltau, Dr. Howard, Dr. L. Feneley, and Dr. Rudolf. Mr. Watson-Williams replied to the very many questions and points raised.

---

At a Committee Meeting held on Thursday, 28th October, 1939, it was resolved to recommend that Dr. L. A. Moore should continue in office as President, together with the present members of Committee, that meetings should be held at such times and places as circumstances might permit, and that all medical men temporarily resident in Bristol and the neighbourhood owing to the necessities of the war should be made temporary members of the Society without subscription, but without being entitled to receive the *Journal* free of charge.

### ℞ Galenicals ℞

SESSION 1939.

OFFICERS.

*Honorary President :* Prof. C. Bruce Perry.

*Honorary Vice-Presidents :* R. V. Cooke, Esq., H. Chitty, Esq., Dr. G. R. Llewellyn.

*President :* Mr. A. G. Farr.

*Honorary Secretary :* Mr. N. F. W. Brueton.

*Honorary Treasurer :* Miss G. M. Higgins.

*Committee :* Miss J. C. Mulcahy, Miss B. F. Thomas, Miss J. Wallace, Mr. A. G. M. Davies, Mr. M. O. Skelton.

THIS year is a not uneventful one in the history of the Society, as the affiliation to the University Union so carefully arranged by the previous Committee has now come into operation. The only major difference which this has made to the Society is that we now hold two meetings a year which are open to all members of the University ; we feel that this arrangement is to be welcomed, as it may help to dispel some of the mystery which surrounds the profession even in educated circles.

The year's programme has resembled those of previous years, consisting of " outside " lectures, a clinical meeting, and some student discussions. In the latter an effort has been made to encourage student effort by holding the meetings in the less formal atmosphere of the General Common Room at the Victoria Rooms, and by devising a scheme of " group attack " of a subject, whereby one member of the group is prepared to discuss one aspect of the subject.

The meetings so far held under this scheme have been very successful, and though their scope is as yet small, they do provide a beginning for the enlargement of student-run activities in the Society. This is one of the main aims of the committee—to make the Society more " alive " by having an increasingly large proportion of its activities run by students.

The year's programme has included a Clinical Meeting held at the Bristol General Hospital, and a visit (by about twenty members) to the R.A.F. Station and Medical Base at Halton. The following lectures have also been given :—

Dr. L. A. Moore, M.B., Ch.B., " **Medicine and the Drama.**"

Sir Arthur Hurst, D.M., F.R.C.P., " **The Physiology and Pathology of the Sphincters.**"

C. A. Joll, Esq., M.S., F.R.C.S., " **Cancer.**"

Dr. L. S. Potter, " **Medicine and the State.**"

Dr. G. L. Foss, " **Some Clinical Uses of the Sex Hormones.**"

In the programme for the coming Michaelmas Term are lectures by Professor Samson Wright and Professor McSweeney on the "**Autonomic Nervous System,**" and by Mr. Eyre-Brooke on "**Amputation Technique.**" There will also be an open debate with the University of Bristol Union.

